# Minimal Residual Disease, Metastasis and Immunity

**DOI:** 10.3390/biom11020130

**Published:** 2021-01-20

**Authors:** Jordi Badia-Ramentol, Jenniffer Linares, Andrea Gómez-Llonin, Alexandre Calon

**Affiliations:** Cancer Research Program, Hospital del Mar Medical Research Institute (IMIM), 08003 Barcelona, Spain; jbadia@imim.es (J.B.-R.); jlinares@imim.es (J.L.); agomez2@imim.es (A.G.-L.)

**Keywords:** CTC, DTC, MRD, dormancy, immunity, metastasis, therapy

## Abstract

Progression from localized to metastatic disease requires cancer cells spreading to distant organs through the bloodstream. Only a small proportion of these circulating tumor cells (CTCs) survives dissemination due to anoikis, shear forces and elimination by the immune system. However, all metastases originate from CTCs capable of surviving and extravasating into distant tissue to re-initiate a tumor. Metastasis initiation is not always immediate as disseminated tumor cells (DTCs) may enter a non-dividing state of cell dormancy. Cancer dormancy is a reversible condition that can be maintained for many years without being clinically detectable. Subsequently, late disease relapses are thought to be due to cancer cells ultimately escaping from dormant state. Cancer dormancy is usually associated with minimal residual disease (MRD), where DTCs persist after intended curative therapy. Thus, MRD is commonly regarded as an indicator of poor prognosis in all cancers. In this review, we examine the current understanding of MRD and immunity during cancer progression to metastasis and discuss clinical perspectives for oncology.

## 1. The Circulating Tumor Cell

At the onset of metastatic dissemination, cancer cells are released from the tumor of origin and intravasate into the bloodstream. These circulating tumor cells (CTCs) have to survive the environmental hazards posed by the circulatory and the immune system to successfully colonize distant organs, thus becoming disseminated tumor cells (DTCs). Invasive primary tumors are constantly shedding CTCs, which are indicative of minimal residual disease (MRD) that may persist after antitumor therapy. Nevertheless, only a small proportion of CTCs are capable of generating distant metastases, which makes metastatic dissemination an extremely inefficient process. Indeed, in a study in 2007, Nagrath and colleagues were able to isolate CTCs from peripheral blood in more than 99% of patients with advanced disease. However, only a subset of positive patients actually developed metastases [1].

Many studies have uncovered processes that may lead to the generation and release of CTCs, with a special consideration to epithelial–mesenchymal transition (EMT) [2,3]. EMT is defined by the loss of epithelial hallmarks, cell–cell and cell–extracellular matrix adhesion features and gain of a mesenchymal-like transcriptional program [4]. EMT can be triggered by the TGF-β, WNT and NOTCH pathways and is mainly regulated by the transcription factors ZEB, SNAIL, SLUG and TWIST1 [5]. EMT is responsible for the acquisition of migratory and self-renewal traits in cancer cells, which are crucial for metastatic dissemination [2,3,5,6]. Indeed, multiple recent studies have reported the existence of stem cell-like CTCs expressing EMT markers such as Vimentin or N-cadherin and which are associated with metastatic potential and poor clinical outcome in various cancers [7,8,9,10,11,12,13].

Evidence suggests that polyclonal clusters of CTCs have increased metastatic potential compared to individual CTCs [14]. Indeed, CTC clustering increases stemness and proliferation by inducing hypomethylation of critical transcription factors such as OCT4, NANOG, SOX2 and SIN3A [15]. Although EMT has been generally linked to the shedding of single cells into the circulation, it can also provoke collective cell migration (CCM) of clusters from primary tumors. Using a model of metastatic intestinal carcinomas in *Drosophila*, Campbell and colleagues showed an association between Sna (the homolog of a human snail) -expressing clones undergoing EMT with clusters of cancer cells, which are able to collectively break through the basal lamina of the gut [16]. Results from this study confirmed previous findings using 3D in vitro models of tumor budding, where cancer cells with a gain of ZEB1 and loss of E-cadherin expression may drive collective rather than single-cell migration [17]. During CCM, single cells that undergo EMT exert mechanical forces that are transmitted throughout the neighboring cells within the cluster [18]. Nevertheless, effective CCM leans on the fact that most cancer cells in the cluster retain epithelial features such as tight junctions, which can be further regulated by Epigen–EGFR signaling occurring in the intercellular nanolumina between neighboring cancer cells [19,20,21,22]. Recent advances point to a key role of the Wnt/planar cell polarity (PCP) pathway driving CCM, where effector Rac1 and ROCK2 are major inducers of cell polarity [18,23,24]. Ultimately, Wnt/PCP activation leads to a RhoA-GTPase-dependent accumulation of RAB13 and NET1 mRNA in cancer cells at the invasive front, and triggers cell migration supported by changes in the ECM and laminin-5 accumulation [25,26,27,28]. Additional research has recently shown that cancer-associated fibroblasts (CAFs) facilitate CCM through heterotypic adhesions and cell-to-cell activation of Wnt and NOTCH pathways [29,30,31,32,33,34].

Once shed into the bloodstream, CTCs must overcome a series of hazards that may compromise their integrity and their capacity to generate distant metastases. Among them, flow speed, turbulence and a high density of circulating objects induce shear stress hindering CTC survival [35]. Individual CTC detachment from the ECM may also induce anoikis that can be overcome by tight junction maintenance in circulating clusters [36]. Several recent studies have shown that hyper-activated platelets adhere to CTC clusters and secrete soluble effectors conferring enhanced survival properties and increased metastatic efficiency to CTCs [37,38,39,40]. In this context, Yu and colleagues described that platelet–tumor cell interaction through Toll-like receptor 4 (TLR4) promotes tumor metastasis [41]. CTCs may also cluster with circulating neutrophils through VCAM1 expression, thus resulting in increased proliferation and migration of CTCs [42,43].

In addition to shearing forces, CTCs are under constant immuno-surveillance, especially from natural killer (NK) cells. Along this line, EMT induces overexpression of NK activator ligands NKG2DL, ULBP1-3 and MHC-I, and downregulation of CADM1, all of which confer increased susceptibility of CTCs to NK-mediated clearance [44,45]. In this scenario, hybrid clusters containing mesenchymal-like and epithelial CTCs are favored against individual CTCs that have undergone EMT, most likely due to NK cells’ decreased sensitivity over cancer cells maintaining epithelial hallmarks [20].

## 2. Mechanisms of CTC Extravasation into Secondary Organs

Adhesion of CTCs to endothelial cells (ECs) is a first key step of the extravasation process. The initial attachment of tumor cells to the vessel endothelium depends on the expression of selectin family receptors by ECs such as E-selectin and P-selectin, which interact with their respective ligands expressed by cancer cells [46]. Selectin ligands include the tetrasaccharide sialyl-Lewis X antigen and its isomer sialyl-Lewis A [47]. Especially Sialyl-Lewis X and P-selectin interaction was shown to contribute to tumor–mesothelial adhesion and metastatic initiation [48]. In addition, CTCs can also locally enhance the expression of selectin through STAT3 pathway activation in ECs, thus increasing adherence to the endothelium [49].

Following attachment, stable adhesion and trans-endothelial migration (TEM) are achieved mainly through integrins and their ligands, including cell-adhesion molecules (CAM) [50,51]. Integrins are heterodimeric adhesion receptors that interact with both ECM and cell–surface ligands [52]. Integrins are key for progression to metastasis, as their decreased expression significantly reduces metastasis initiation [36]. During TEM, α3β1 and α6β1 integrins contribute to tumor cell adhesion to the sub-endothelial basement membrane in multiple cancers [53]. In breast cancer, for example, CTCs may undergo β1 integrin-dependent adhesion and TEM by inducing vascular endothelium remodeling [54]. In addition, Aragon-Sanabria and colleagues proposed that intercellular adherent junctions’ disassembly and endothelial cell contractility were necessary for endothelial barrier disruption, both being modulated by cancer cells originating signaling [55]. For instance, cancer-cell-secreted SPARC facilitates EC junction opening, thereby contributing to vascular permeability and paracellular TEM [56]. In this context, it has also been demonstrated that secreted factors from cancer cells may trigger focal adhesion protein (FAK)/E-selectin cascade in ECs to induce formation of vascular hyper-permeability foci in pre-metastatic lungs ECs [57].

As mentioned in the first section, CTCs may interact with platelets and leukocytes during metastatic dissemination. Interestingly, while a majority of CTCs are associated with different platelet coverage [58], cancer cell and platelet interactions mediate CTCs adhesion to ECs, thereby promoting tumor metastasis [46]. In addition, platelets may secrete factors contributing to vascular permeability [59] and cancer cell extravasation [60]. In this context, platelet-secreted TGF-β induces cancer cells EMT, thus increasing their invasiveness and their ability to extravasate [61].

Alternatively, physical interplay between leukocytes and CTCs may also increase CTCs’ capacity to adhere to the endothelium [58]. For instance, neutrophils increase tumor cell adhesion to hepatic sinusoidal ECs [62]. Moreover, neutrophils expressing β2 integrin may also enhance CTC-EC adhesion by interacting with ICAM1 expressed by cancer cells and ECs in breast cancer [63]. Following initial adhesion, the interaction between αMβ2 integrin on neutrophils and ICAM1 on breast CTCs induces cancer cell TEM [64]. Finally, macrophages may also interact with CTCs to further assist them during extravasation [65]. For instance, macrophages may secrete tumor necrosis factor α (TNF-α), which induces E-selectin expression by endothelial cells [66]. In this context, metastatic tumor cells may increase TNF-α secretion by Kupffer cells, thus leading to E-selectin, VCAM1 and PECAM1 expression in hepatic sinusoidal vessels [67].

## 3. Organotropism and Metastatic Niche

Organ specification for metastatic seeding can be determined by physical proximity to the tumor of origin. For example, L1CAM+ metastasis-initiating cells originating from colorectal carcinomas are more prone to invade the liver through portal circulation [3,68,69,70]. However, the determinants of metastatic site specification remain an open field of research, and alternative hypotheses suggest that CTCs may need intrinsic traits in order to thrive into specific metastatic loci. Several mechanisms depending on the expression of miR-105, L1CAM, MMP-2, COX2 and HB-EGF have been described for brain metastasis initiated by breast and lung originating CTCs [71,72,73]. Alternatively, expression of CXCR4 is required by breast cancer cells to metastasize into the bone [2,73], while lung metastasis originating from primary melanoma and breast tumors depends on ANGPTL4 and SPARC expression [56,74]. In addition, colorectal cancer (CRC)-derived metastasis grown in the liver can further metastasize to the lung through downregulation of p38 MAPK signaling and expression of PTHLH [75].

In order to successfully initiate metastases, DTCs must harbor stem cell-like properties of self-renewal and proliferation typical of “cancer stem cells” (CSCs). For instance, tumor-initiating cells are characterized by bona fide expression of normal stem cell markers such as LGR5 and EPHB2 in CRC [70,76,77,78,79]. Similarly, single cell analysis has shown that cancer cells in nascent metastases from breast primary tumor express a stem cell-like genetic program [80]. Therefore, DTCs may require paracrine signaling originating from the local environment to maintain stemness properties. In this aspect, the “seed and soil” hypothesis proposed by Stephen Paget in 1889 suggests that the foreign niche is key to metastatic initiation [81]. As a matter of fact, several studies have described that local and bone-marrow-derived cells are recruited during the colonization to provide factors activating stem-cell pathways such as TGF-β, BMP, Wnt, Notch and Stat3 in DTCs [2]. Alternatively, growing evidence supports the hypothesis of a pre-metastatic niche (PMN) assembled prior to organ colonization and controlled by primary tumor-secreted factors like exosomes. As reviewed elsewhere, exosomes may influence virtually every component of the PMN [82,83,84,85].

Fibroblasts are one of the key cellular components of the metastatic niche, where they are activated into cancer-associated fibroblasts (CAFs) by TGF-β [86,87,88,89], FGF2 and members of the PDGF family [89]. Alternatively, IL-1⍺ and IL-1β secretion by metastasis-initiating breast cancer cells [90] may activate CAFs to secrete ECM-remodeling factors such as matrix metalloproteinases (MMPs), LOX enzymes, Periostin, Tenascin-C and Versican, among others, thus providing a favorable niche for organ colonization [82,89,91]. Additionally, CAFs provide a niche factor that maintains stem cell properties of metastatic initiating cells, including but not limited to CXCL9, CXCL10 [90], CXCL12 [92], IL-11 [86], TGF-β1 [93], HGF [94], CCL18, Osteopontin and plasminogen activator inhibitor 1 [95]. Interestingly, several studies have reported the existence of several CAF subpopulations with functional differences coexisting within a tumor, including defined populations that promote stemness. For instance, αSMA+ CAFs in oral cancer are positively correlated with the presence of CSCs [96]. Similarly, NF-κB expression in CD10+GPR77+ CAFs from breast and lung cancers maintains stem cell properties of cancer cells through secretion of IL-6 and IL-8 [97]. Finally, both CAFs and metastatic initiating cells may recruit immune cells such as myeloid-derived suppressor cells (MDSCs), macrophages and neutrophils, which in turn support CTC survival and organ colonization by establishing a permissive immunosuppressive environment [98,99,100,101,102,103,104,105].

## 4. The Metastatic Dormant Cell

**Figure 1:**
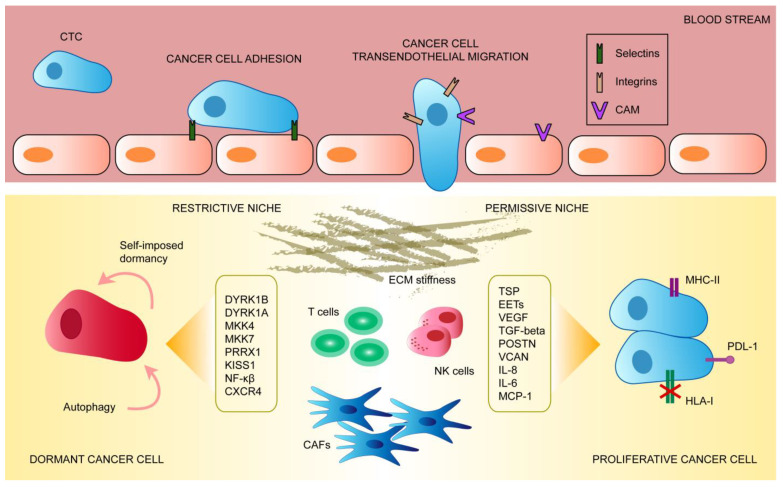
In order to colonize distant organs, circulating cancer cells will eventually adhere and transmigrate through the endothelium thanks to the expression of different ligands and receptors. Following trans-endothelial migration, cancer cells will either enter a dormant or a proliferative state. Disseminated cancer cells’ dormancy or proliferation is determined by both intrinsic signaling and cues originating from the surrounding restrictive or permissive niche.

Tumor dormancy refers to a transient growth and mitotic arrest occurring in cancer cells (see Figure 1). This phenomenon is critical for the establishment of a resistant MRD that may lead to tumor relapses years after therapy [106,107]. Dormancy that usually occurs during the formation of tumors or after dissemination to distant organs enables DTCs to adapt to and colonize distant organs [108]. In this sense, several distinct mechanisms have been proposed to maintain single cell dormancy and dormant micrometastases, which include angiogenic dormancy, intrinsic or extrinsic cellular dormancy and immune-mediated dormancy [109].

The expansion of micrometastatic lesions is at first restricted by similar rates of cancer cell proliferation and apoptosis, primarily due to poor vascularization. Several studies have reported that molecules such as thrombospondin (TSP), vascular endothelial growth factor (VEGF) and epoxyeicosatrienoic acids (EETs) expressed by ECs in the stable microvasculature might influence tumor angiogenesis, which consequently regulates the maintenance of DTCs dormancy or their switch into a proliferative state [110,111,112,113]. Similarly, the recruitment of vascular and myeloid cells by secreted tissue factor (TF) has been shown to modulate cancer cells dormancy in glioma cells [114].

DTCs can also activate self-imposed intrinsic dormancy programs (see Figure 1), for example, by F-box/WD repeat-containing protein 7 (FBXW7) and the leukemia inhibitory factor receptor (LIFR), which allow them to adapt to new microenvironments and remain unaffected by therapies targeting highly proliferative cells [115,116]. DTCs can also alter signaling pathways that coordinate metabolic homeostasis, such as the PI3K-AKT pathway [117,118]. In addition, autophagy-related 7 (ATG7) has also been involved in survival of dormant breast cancer cells [119]. Finally, latent breast and lung carcinoma cells may express stem cell-like SOX transcription factors, which self-impose a slow proliferating state [120].

Alternatively, cellular dormancy may occur through extrinsic mechanisms (see Figure 1), and the differential ability of organs to support DTC growth has led to the classification of microenvironments as dormancy-permissive or dormancy-restrictive [121,122,123]. Indeed, local microenvironment-originating stress signals have been proposed to induce DTC dormancy, including specific kinases such as dual specificity tyrosine phosphorylation-regulated kinase 1B (DYRK1B) and 1A (DYRK1A), mitogen-activated kinases such as MKK4 (MAPKK4) and MKK7, paired-related homeobox transcription factor (PRRX1), kisspeptin 1 (KISS1), downregulation of the C-X-C motif chemokine receptor 4 (CXCR4) and the activation of the canonical nuclear factor kappa-light-chain enhancer of activated B cells (NF-κB) pathway [124,125,126,127,128,129,130,131,132,133,134,135]. Moreover, early studies showed that ECM protein fibronectin (FN) may determine whether DTCs can remain in a dormant state by interacting with the urokinase plasminogen activator receptor (uPAR) in cancer cells. Indeed, high levels of uPAR increase adhesion of cells to fibronectin and generate persistent activation of ERK, which is necessary for tumor growth [136,137].

Finally, DTCs dormancy may also depend on an equilibrium state with the immune surveillance system [138], exerted mainly by tumor-infiltrating lymphocytes (TILs) and NK cells and mainly regulated by the secretion of interferon gamma (IFNγ) [138,139,140,141]. In this setting, the immune system may regulate DTC numbers and micrometastatic lesion size [142]. For instance, an increased density of immune cells has been observed in the bone marrow of patients with breast cancer displaying dormant DTCs [143]. In this line, CD4+ and CD8+ T cell depletion in vivo resulted in cancer cells escaping from dormancy [120,144]. Furthermore, the action of NK and T cells can be regulated by tumor cells on the basis of HLA class I expression. Therefore, variations in the expression of these proteins in DTCs are key to NK and T cells’ cytotoxic response and dormancy maintenance [145,146].

## 5. Escape from Dormancy and Immune Evasion

Escape from dormancy is often directed by intrinsic changes of gene expression patterns in DTCs. Alternatively, it may also depend on certain traits and interactions with the host tissue (see Figure 1). The secretion of soluble factors, including interleukin-8 (IL-8) and monocyte chemoattractant protein-1 (MCP-1) by cancer cells has been shown to promote breast cancer cells proliferation [147]. Upregulation and activation of the vascular cell adhesion protein 1 (VCAM1) and periostin (POSTN) may induce escape from dormancy [112,148]. In addition, increased matrix stiffness related to TGF-β pathway activation may also reinstate cell proliferation [149]. TGF-β, which controls the expression of the inhibitor of differentiation family of proteins (ID), has been reported to promote metastasis and regulate the dormant state of cancer cells. In this line, increased ID1 and ID3 expression in basal or triple-negative breast tumors reactivates and sustains cancer cell proliferation in the lung during metastatic colonization [150,151]. On the other hand, Coco, a secreted antagonist of TGF-β ligands, has been reported to facilitate escape of dormancy in disseminated breast cancer cells by blocking BMP signaling, thus inducing lung-specific colonization [152]. Additionally, disseminated prostate cancer cells in the bone marrow have been shown to escape their dormant state by downregulating TGF-β2 expression and activating its downstream target myosin light chain kinase (MLCK) [153].

Eventually, environmental pressure will select for metastatic cells able to escape the cytotoxic activity of TILs, a process denominated as “immunoediting” [138,139,154,155]. As reviewed in the 2011 update of the hallmarks of cancer, cancer cells co-opt inflammatory cells to assist their expansion, while evading antitumor activity of TILs [156]. However, how nascent metastases evade antitumor immunity still needs to be fully addressed. Here we revisit four global strategies that metastatic initiating cells may adopt to escape antitumor immunity and grow into full-blown metastases.

First, dormant cells as well as slow-cycling cancer cells may evade direct recognition from TILs ab initio. DTCs are stochastically primed to grow into full-blown metastasis, which is prevented by NK-mediated immunosurveillance. Indeed, when NK cells are depleted using NK-specific monoclonal antibodies, the amount of lung metastases increases in a mouse model of latent metastatic breast cancer [120]. Consequently, SOX9-expressing dormant cancer cells impair NK cytotoxic activity by downregulating ULBP activators of NKG2D receptors on NK cells [120,157]. In a clinical study, downregulation of ULBP1 was detected in CTCs and might be a result of EMT [158]. Alternatively, CD8+ T cell recognition could be avoided by masking antigenic presentation. In a study using T cells engineered to detect and kill GFP-expressing cells (JEDI cells), Agudo and colleagues showed that quiescent Lgr5-GFP+ adult skin stem cells may evade T cell recognition by downregulating expression of MHC-I, a mechanism that could also apply to quiescent/dormant cancer cells [159].

A second mechanism involves the generation of a permissive, anti-inflammatory PMN prior to organ colonization, with cells exerting immunosuppressive roles such as macrophages, myeloid-derived suppressor cells (MDSCs), and neutrophils [85,98,160]. Supporting this possibility, a recent study evidenced that immune evasion may already occur in lung premalignant lesions [161], where circulating IL-6 induced macrophage polarization into an immunosuppressive state and decreased TIL infiltration through the STAT3 pathway [162]. In this context, CAFs may participate to suppressing antitumor immunity by different means, including secretion anti-inflammatory cytokines [89,99,163,164,165], modulation of ECM stiffness [166,167,168] and direct inhibition of CD8+ TILs through MHC-II antigen presentation [169,170]. Of note, CAFs are the main producer of IL-6 in primary tumors and may be a potential source of circulating IL-6 programming distant immunosuppressive PMN [97,163,171,172].

As a third mechanism, the activation of certain oncogenes in DTCs could trigger the secretion of anti-inflammatory cytokines, which dampens antitumor immunity. This process has been termed as the “intrinsic pathway” of cancer immunity [173,174]. In this context, TGF-β is probably the most prominent anti-inflammatory cytokine, which exerts a plethora of immunosuppressive effects on the tumor immune microenvironment [175]. For instance, TGF-β may be secreted by metastatic initiating cells in order to modulate T cell exclusion in CRC, consequently allowing efficient metastatic initiation by avoiding TIL immunosurveillance [86,88,176]. Furthermore, TGF-β mediates expansion of regulatory T cells (Treg), which in turn impairs antitumoral response [175].

A final mechanism may imply the expression of immune checkpoint ligands by DTCs, for example, PD-L1, the ligand of the programmed death receptor 1 (PD-1). The PD-1/PD-L1 pathway is one of the most well-understood inhibitory mechanisms of adaptive immunity. While PD-1 is expressed almost exclusively by lymphocytes, PD-L1 expression can be triggered by TIL-secreted IFNγ in multiple cell subtypes of the tumor microenvironment, including the cancer cells [177,178,179,180,181]. Primary tumors with high PD-L1 expression are likely to generate PD-L1+ distant metastases [145], which implies that CTCs may already present surface expression of PD-L1 and resistance to TIL-mediated immune surveillance [182,183,184,185,186,187]. Indeed, expression of PD-L1 in EMT-like CTCs predicts reduced survival in patients with non-small cell lung cancer [188]. Metastatic initiating cells can also co-opt CTLA-4 immune checkpoint to avoid T cell activation—for instance, TGF-β-induced CSC expression of CD80, leading to T cell inhibition through the CD80-CTLA-4 axis in pre-clinical models of squamous cell carcinoma [189].

## 6. Clinical Perspectives

Cancer therapy has experienced significant improvements during recent decades. However, advanced cancer, especially metastatic disease, remains extremely difficult to cure. As discussed above, CTCs or dormant DTCs persist often undetected as MRD after treatment and represent a threat of relapse and metastasis formation. Furthermore, the number of resistant CTCs increases after therapy [9,33]. Therefore, early detection of MRD has become a priority for monitoring cancer progression and preventive therapy.

Currently, MRD can be assessed by detecting CTCs and/or circulating tumor DNA (ctDNA) in peripheral blood by performing liquid biopsies (LB), which are far less invasive than biopsies from solid tumors. Hence, new techniques are being established to accurately detect CTCs and CTC clusters in plasma [187,190,191,192]. A better understanding of CTCs could lead to more accurate monitoring of tumor responses and patient stratification [13,193]. In this regard, modelling metastatic dissemination using patient-derived xenograft (PDX) mouse models can elucidate the heterogeneity of CTCs and their possible outcomes in patients [12,194]. Indeed, detection of CTC clusters and stem-like CTCs is correlated with decreased disease-free survival [13,33,185,195]. Alternatively, ctDNA released by apoptotic cancer cells including CTCs and DTCs can be detected thanks to their specific cancer-associated mutation pattern. In recent clinical studies, almost all patients with MRD from CRC had detectable levels of ctDNA in the blood stream. In this context, presence of ctDNA was correlated with a worse outcome [196,197]. Early detection of MRD by following CTCs and ctDNA in LB will provide valuable tools for decision-making and clinical management of cancer patients.

Immunotherapy based on blocking antibodies against immune checkpoint receptors and ligands, including PD-1/PD-L1, CTLA-4 and NKG2A, is of particular interest in the clinical setting [187,198,199,200,201,202,203]. Immune checkpoint inhibitors (ICI) have proven effective in “immunologically hot tumors”, such as melanoma and lung cancer [204], which are densely infiltrated with lymphocytes [205]. In opposition, treatment with ICI of immune-excluded “immunologically cold tumors”, which encompass most solid tumors, is still challenging. In this regard, the TGF-β has arisen as a likely cause for the immune exclusion observed in cold tumors. In mouse models of colorectal and urothelial cancers, combination of TGF-β-pathway inhibitors with ICI leads to an amplification of antitumor immune responses, whereas ICI alone typically fail [168,176,206]. Additionally, recent clinical trials have shown that standard-of-care procedures such as radiotherapy, chemotherapy and oncogene-targeted therapies might raise antitumor immunity, thus leading to improved survival in patients when ICI are added [207,208,209,210,211]. As discussed above, immune evasion from NK and T cell surveillance characterizes all steps of efficient metastatic progression, from CTC release to the generation of overt metastases. Indeed, CTCs, DTCs and metastatic initiating cells take advantage of the expression of checkpoint ligands such as PD-L1 to escape TIL antitumor activity in a similar fashion to macrometastases. Thus, ICI-based anticancer strategy may be especially relevant against MRD.

The spectrum of responses to immunotherapy has underscored the relevance of immunologic biomarkers to predict benefit from therapy [205,212]. Perhaps one of the best examples is a phase II clinical trial designed to treat multiple solid tumors with Pembrolizumab, an anti-PD-1 antibody. This trial demonstrated that patients bearing tumors with microsatellite instability (MSI), which correlates with increased lymphocytes infiltration, were responding better to treatment when compared to patients with microsatellite stable (MSS) tumors [213]. With few exceptions, an increased density of TILs, particularly CD8+ cytotoxic T cells, is correlated with better responses to ICI and increased overall survival [214,215,216,217,218]. This trait could be exploited to create new diagnostic tools predicting patient response to treatment and guiding decision-making [219,220]. In this line, recent studies have shown that treatment with ICI enhances a peripheral T cell response correlated with increased TILs [221,222,223,224,225]. This finding opens the possibility of early patient stratification by detecting relevant immune populations in LB [224,226].

In conclusion, a better understanding of MRD will pave the way for a more accurate and personalized management of anticancer therapies. It is worth noting that several clinical trials are currently investigating the predictive power of ctDNA, CTCs, soluble factors and peripheral immune populations for cancer progression and response to therapy (see Table 1). Given its limited invasiveness, repeated LB enables dynamic monitoring of MRD and may potentially help to foresee metastatic initiation.

## Figures and Tables

**Table 1:** Clinical trials currently exploring liquid biopsy as a source of novel biomarkers of cancer progression and treatment. CTCs = circulating tumor cells; ctDNA = circulating tumor DNA. Source: https://clinicaltrials.gov.

Identifier	Diagnostic Parameter	Disease	Objective	Starting Year
NCT02072616	CTCs	Pancreatic adenocarcinoma	Determination of the diagnostic accuracy of the combined detection of CTCs and circulating tumor DNA for the diagnosis of pancreatic adenocarcinoma	2014
NCT04239105		Breast neoplasms	Establishment of the Raman Spectrum Device for CTCs detection and to analyze fluctuations of CTC numbers during chemotherapy and neoadjuvant chemotherapy.	2020
NCT02827344		Non-small cell lung cancer	Feasibility of the analysis of PD-L1 expression on CTCs and evolution of the percentage of PD-L1-expressing CTCs prior and after receiving immunotherapy.	2016
NCT03213041		Metastatic breast cancer	Evaluation of the clinical impact of treatment with pembrolizumab and carboplatin by detecting and measuring CTCs.	2017
NCT02812680		Esophageal cancer	Use of CTCs as predictive biomarkers for neoadjuvant therapy using CTC chips.	2016
NCT04367311	ctDNA	Lung cancer	Assessment of ctDNA clearance to determine responses to adjuvant chemotherapy + Atezolizumab.	2020
NCT04148066		Non-small cell lung cancer	Prediction of patients with cancer cell clones resistant to Osimertinib and Crizotinib by ctDNA detection.	2019
NCT04353557		Breast cancer	Post-operative kinetics and association with time to recurrence of detected ctDNA.	2020
NCT03926260		Metastatic non-small cell lung cancer	Identification of early patient response to treatment by detecting changes in ctDNA concentration.	2019
NCT04259944		Colon cancer	Use of fluctuations of ctDNA concentration for patient allocation to receive Capecitabine, CAPOX or FOLFIRI after surgery.	2020
NCT04135079	Peripheral leukocytes	Multiple myeloma	Evaluation of immune transcriptome profile from peripheral blood mononuclear cells by RNAseq and CyTOF and cytokine profiling.	2019
NCT04127864		Colorectal cancer	Determination of alterations of cytokine profiles in blood from patients in response to surgery.	2019
NCT03493581		Non-small cell lung cancer	Immune profiling of peripheral monocytes, B, T, NK and dendritic cells to assess resistance to anti-PD-1 immunotherapy.	2018
NCT04464122		Neuroendocrine tumors	Immune profiling for diagnosis and evaluation of response to chemotherapy in locally advanced or metastatic cancer.	2020

## Data Availability

All data presented in this review is freely available.
